# Predictors of dieting and non-dieting approaches among adults living in Australia

**DOI:** 10.1186/s12889-017-4131-0

**Published:** 2017-02-20

**Authors:** Stuart Leske, Esben Strodl, Xiang-Yu Hou

**Affiliations:** 1 School of Psychology and Counselling, Queensland University of Technology, Victoria Park Road. Kelvin Grove, Brisbane, QLD 4059 Australia; 2 School of Public Health and Social Work, Queensland University of Technology, Victoria Park Road. Kelvin Grove, Brisbane, QLD 4059 Australia

**Keywords:** Dieting, Non-dieting, Theory of planned behaviour, Self-efficacy, Identity theory, Weight control beliefs, Non-planning, Self-identity, Locus of control

## Abstract

**Background:**

There is a dearth of research comparing why dieting and non-dieting approaches are adopted. A greater understanding of reasons underlying dieting and non-dieting attempts will help to identify target beliefs for interventions to support and motivate adults to attempt whatever approach they are willing and/or able to pursue. We investigated the predictors of dieting and non-dieting approaches in Australian adults using predictors that were identified in a previous qualitative study.

**Methods:**

We conducted a prospective study, with two waves of data collection occurring 4 weeks apart. At baseline, participants completed a questionnaire assessing constructs drawn from the theory of planned behaviour (attitude, subjective norm, and self-efficacy), past behaviour, non-planning, attributions for dieting failure, weight control beliefs, and dieting and non-dieting intentions. We used path modelling to analyse responses.

**Results:**

At baseline, 719 adults (52.2% male) aged between 18 and 76 completed the questionnaire. Four weeks later, 64% of participants (*n* = 461) reported on their dieting and non-dieting behaviour in the past month. Past behaviour, attitude, subjective norm, and self-identity significantly predicted dieting intentions. Dieting intentions and past behaviour significantly predicted dieting behaviour, while non-planning and self-efficacy did not. The model explained 74.8% of the variance in intention and 52.9% of the variance in behaviour. While most findings were similar for the non-dieting model, subjective norms and self-identity did not predict intention, while self-efficacy and self-identity both predicted non-dieting behaviour directly. The non-dieting model explained 58.2% of the variance in intention and 37.5% of the variance in behaviour.

**Conclusions:**

The findings from this study provide support for the application of TPB and identity theory constructs in the context of both dieting and non-dieting behaviour. Self-efficacy and self-identity appear more relevant to non-dieting behaviour than dieting behaviour, while subjective norms was more influential in predicting dieting. Practitioners wishing to encourage either approach in their clients should attempt to modify the constructs that influence each approach.

**Electronic supplementary material:**

The online version of this article (doi:10.1186/s12889-017-4131-0) contains supplementary material, which is available to authorized users.

## Background

Obesity has been labelled a global epidemic by the World Health Organisation, with worldwide rates nearly doubling since 1980 [1]. While obesity increases the risk of asthma, cardiovascular diseases, chronic back pain, gallbladder disease, osteoarthritis, some cancers, and type II diabetes [2], there is evidence suggesting that the risk of these diseases can be reduced through modest weight loss and maintenance in overweight and obese adults [3].

Dieting, which can be defined as “the intentional and sustained restriction of caloric intake for the purpose of reducing body weight or changing body shape” [4], has historically been the main treatment paradigm for individuals with excess weight. A previous meta-analysis suggested that there is little support for the idea that dieting in isolation results in lasting weight loss or health benefits [5]. While short term moderate weight loss can be achieved in multicomponent programs [6], it is often regained over time, although some studies lack the long-term follow-up necessary to document this weight regain [7]. Nonetheless though the magnitude of weight loss and weight loss maintenance potentially may be small, without interventions, individuals tend to continue to gain weight over time [8].

During the 1990s, the dieting or weight-centred paradigm was criticised for its inability to achieve long-term weight loss [9, 10]. More recently criticisms of this paradigm have also included that it contributes to food and body preoccupation, eating disorders, lower self-esteem, weight cycling and weight stigmatisation [11–13]. In response an alternative non-dieting paradigm has emerged which focuses instead on body acceptance, and health behaviours and outcomes, without a focus on weight loss [14]. Other non-dieting tenets included eating intuitively (i.e., relying on internal hunger and satiation cues) rather than dietary restriction [15], and enjoying physical activity in contrast to participating in structured exercise [14]. A review of six randomised controlled trials involving non-dieting approaches found clinically and statistically significant improvements in blood lipids, blood pressure, body image, eating disorder pathology, physical activity, mood, and self-esteem [14]. No weight gain was observed following any of the non-diet interventions. Given that approximately 30–40% of weight-concerned adults report trying to manage weight using what may be regarded as a diet [16, 17], a non-dieting intervention is potentially an alternative approach for those interested in health but not interested in weight loss.

Currently little is known about why individuals choose to adopt non-dieting approaches, and how this is similar to or different from the reasons why individuals choose dieting approaches. A greater understanding of this will help to identify target beliefs for interventions to support or motivate adults to attempt whatever approach they are willing and/or able to pursue. This study compared and contrasted predictors of dieting and non-dieting to provide a greater understanding of what constructs uniquely predict each approach and how an individual might transition from one approach to another.

### Literature review

To address the dearth of research comparing and contrasting these approaches in an adult Australian population, this study aimed to empirically test the association between choosing a dieting or a non-dieting approach and a range of psychological constructs that had been associated with this choice in a previous qualitative study we conducted [18]. Because there appeared to be a strong overlap between some of the constructs identified in this qualitative study and the Theory of Planned Behaviour (TPB), this study involved a core TPB methodology supplemented by additional psychological constructs from the qualitative study and wider literature. These constructs included identity [19], influential others [20], outcome expectations [21, 22], attributions for dieting failure [18, 20], non-planning and self-efficacy [18], and beliefs about whether to focus on weight or lifestyle [18, 23].

### Theory of planned behaviour

The Theory of Planned Behaviour (TPB) is a social cognitive theory comprising attitudes, subjective norms and perceived behavioural control. Attitudes are considered to be positive or negative evaluations of behaviour; subjective norms are thought of as perceptions of social pressure to perform behaviour; while perceived behavioural control refers to a general perception of control over behaviour. These concepts purportedly influence intentions and intentions influence behaviour.

In the context of dietary behaviour, the TPB has been used previously to predict consumption of a low-fat diet [24, 25] and dieting [26–28]. In the dieting studies intention has emerged as the only predictor of dieting behaviour [26], while attitudes, prior dieting, indirect perceived control (beliefs underlying the direct measure of perceived behavioural control), and descriptive norms (perceptions of what others do) predicted dieting intentions and dieting behaviour [27, 28]. A recent meta-analysis of the TPB’s application to the prospective prediction of health behaviours suggested that the theory more efficaciously predicted dieting behaviour than risk detection, safe sex, and drug abstinence behaviours [29].

### Self-identity

Self-identity has been proposed as a useful addition to the TPB [30]. Originating from identity theory [31, 32], self-identity is conceptualised as an individual’s salient aspect of self that is related to certain behaviour [33]. These roles are conceptualised with reference to wider society [34], meaning that self-identity can reflect several social roles that individuals fulfil [31, 32]. Stryker proposes that individuals behave in a manner consistent with their identity, progressively more so as this identity becomes more salient. Self-identity has previously predicted dieting intentions [35], low-fat diet consumption [24], low animal fat diet [36] and actual healthy eating behaviour [37]. A recent meta-analytic review of the inclusion of self-identity in TPB studies [30] supports its additive effects; regression analyses indicated that self-identity explained on average an incremental 6% of the variance in intention after controlling for the TPB constructs.

### Impulsivity

Two recent studies have augmented the TPB by including impulsivity-related measures capturing non-reflective information processing. In two prospective studies, impulsivity significantly predicted avoidance of snacking after TPB constructs were controlled for, explaining an additional 10% [38] and 6.9% [39] of variance and an additional 6.9% of variance. Individuals higher in impulsivity were more likely to snack in both studies. The relevance of impulsivity to dieting behaviour is evident in qualitative studies suggesting that diets are adopted with a quick fix in mind [20] while non-dieting approaches appear to be adopted with a longer-term perspective in mind [18].

### Locus of control measures

Based on previous qualitative studies [18, 20], domain-specific locus of control beliefs concerning diet adherence and weight control appeared relevant to predicting dieting and non-dieting behaviour. Originating from Rotter’s social learning theory [40–42], locus of control describes the degree to which individuals perceive they can control events. These perceptions are either internal (contingent on one’s behaviour or characteristics) or external (determined by luck, chance, fate, or the environment) [43]. Failing to adhere to a diet might be internal (e.g., lacking discipline), external (e.g., diet expense or restrictiveness), or somewhere along this continuum. Previous reasons for diet nonadherence include the diet being “unrealistic”, “unsustainable”, or “too expensive”, although self-blaming has also been noted [20]. It has been observed qualitatively that blaming diets precipitated non-dieting attempts [18]. Accordingly, we measured attributions for dieting nonadherence in this study.

Weight control beliefs can also distinguish between dieters and non-dieters [18, 23]. Laliberte and colleagues [23] have conceptualised these as “beliefs that you can and should control your weight” (BCWeight) and beliefs that “you should strive for a healthy lifestyle and accept the resulting weight” (BCLifestyle). Both items represent a high internal locus of control – with BCWeight presumably underpinning a dieting approach, and BCLifestyle presumably underpinning a non-dieting approach. Strong endorsers of weight control beliefs were more likely to be involved in restricted eating and binge eating, as well as also distinguishing between patients with eating disorders and non-patients [23]. Conversely, individuals in a non-clinical sample strongly endorsing beliefs in a healthy lifestyle had a lower risk of both restrained and binge eating.

### Aims and hypotheses

In light of the literature above, we had one main research question: (1) What are the determinants of dieting and non-dieting intentions and behaviours? Drawing from the TPB model, we hypothesised that more positive attitudes towards dieting (attitudes), more perceived social pressure to diet (subjective norms) and more confidence in dieting (self-efficacy) would predict dieting intentions and dieting behaviour. We hypothesised that non-dieting attitudes, non-dieting subjective norms and non-dieting self-efficacy would predict non-dieting intentions and non-dieting behaviour. Although we measured perceived control, it was omitted due to low internal reliability (< .60), the salience of self-efficacy in our qualitative findings [18], previous literature [24, 25] and the presence of other domain-specific measures addressing internal and external control (weight control beliefs, dieting adherence beliefs).

For self-identity, we hypothesised that seeing oneself as the type of person who diets, is concerned with their weight, or sees themselves as overweight would predict dieting intentions and behaviour, while seeing oneself as someone who eats healthily without dieting would predict non-dieting intentions and behaviour. We hypothesised a direct link between self-identity and behaviour based on previous research [44] and theoretical predictions that identity may represent more impulsive, unplanned routes to engaging in a behaviour [45, 46]. Based on our previous findings, we hypothesised that scoring higher on the impulsiveness sub trait of non-planning reflecting “present orientation” or “lack of futuring” [47] would positively predict dieting intentions and behaviour, while scoring lower would negatively predict non-dieting intentions and behaviour. We expected that individuals high in non-planning impulsiveness would be inclined to quickly form intentions to diet to achieve short-term goals. We reasoned that there might be a direct link between non-planning and behaviour through a more spontaneous pathway. Specifically, individuals’ weight loss goals, coupled with widespread marketing of structured diets promoting weight loss [48], could result in impulsive adoption of a diet without deep consideration. Given our prospective design, some adults may not have formed an intention to diet at baseline yet decided to diet after completing the baseline questionnaire. Because some diets have extensive, well-designed plans available online [49], these diets would suit someone high on non-planning who felt unable or unwilling to plan themselves. Conversely, we considered eating healthy without dieting a more deliberative behaviour adopted with long-term goals in mind and so we speculated that this would be intended and actually adopted by individuals lower in non-planning impulsiveness. For dieting adherence, we hypothesised that attributing failure of a diet to oneself would reinforce dieting intentions, while attributing dieting failure to the diet would predict non-dieting intentions. We expected that BCWeight would positively predict dieting intentions while BCLifestyle would positively predict non-dieting intentions.

## Methods

### Participants

We required that participants were aged 18 and over and living in Australia. Because most non-dieting studies have included overweight or and obese female participants [50], some recruitment strategies focused exclusively on males. Because normal weight adults also attempt to lose weight [51], they were eligible for inclusion in this study since the topic of dieting was evidently applicable to them and so they could serve as a reference group for overweight and obese adults in the analyses.

### Procedure

We obtained ethical clearance for the study from the Queensland University of Technology’s Human Research Ethics Committee. We recruited a community-based sample through snowball sampling, media releases, articles in newsletters and newspapers, notices at the local health clinic, radio broadcasts, public venues (voting hall on election day), and the newsletters of various small businesses with an interest in food and health. Snowball sampling was implemented to access community members not affiliated with universities and to ensure that the sample size obtained was sufficiently powered for the planned statistical analyses. Prior to participating online, participants viewed an information sheet informing them of the voluntary nature of consent and outlining their right to withdraw from the study at any time. Participants were subsequently informed that beginning the questionnaire indicated that they had consented to taking part in the study.

We used a prospective shortitudinal study design to enable temporal ordering and conclusions about prediction to be drawn. Australian adults subsequently completed a baseline questionnaire (see ‘Additional file 1: questionnaires.docx’) consisting of a demographics section, items assessing predictors derived from the TPB (attitude, subjective norm, and self-efficacy), and items assessing self-identity, attributions for dieting failure, weight control beliefs, and non-planning. Four weeks later, the Time 2 questionnaire (see ‘Additional file 1: questionnaires.docx’) assessed participants’ self-reported dieting and non-dieting behaviour in the past month. Time 1 and Time 2 questionnaires were matched via a code identifier. At Time 1 and Time 2, participants were given the opportunity to enter a prize draw to win one of three iPad Minis, each valued at AUD$315. We collected data between June and November 2013.

### Measures

#### Dependent variables

The target behaviours, ‘dieting’ and ‘non-dieting’, were both adapted from a definition from the National Task Force on the Prevention and Treatment of Obesity [4] in their review of the behavioural effects of dieting in obese adults. Items were developed with the same level of specificity in terms of the target (weight loss and/or changing body shape), action (dieting), time (in the next month), and context (in any situation), as recommended by Fishbein and Ajzen [52]. We did not state specific durations for the behaviours because we considered that these items measured attempts at each approach (e.g., [24]), and so we were unconcerned with adherence. We specified that dieting is:

“Intentionally restricting your calorie intake (energy or kilojoules derived from food) or increasing certain type of foods (e.g., high carbohydrate or high protein) to lose weight and/or change your body shape.”

We added the phrase “increasing certain types of foods (e.g., high carbohydrate or high protein)” since some weight loss diets (e.g., the Paleo Diet) are based on eating certain types of foods to promote satiety without restricting or counting calories. Participants were further instructed that dieting did not include fasting for religious purposes, since this was not a weight loss attempt. We further clarified that dieting could include any eating program planned by someone else, or any approach an individual had designed on their own, to use in any context, so long as participants intended to lose weight and/or change their body shape. Participants were provided with examples of popular diets (e.g., The Paleo Diet or The Pritikin Diet), commercial weight loss programs (e.g., Weight Watchers or Jenny Craig), or meal replacements (e.g., Tony Ferguson or OptiSlim). This broad operationalisation was necessary to ensure that participants were certain whether they were dieting or non-dieting, because we could not clarify this definition once participants began the survey online. A clear definition was important since one-item unambiguous questions assessing dieting are more strongly associated with reported energy intake than more general single-item questions (e.g., “doing anything to lose weight”) [53].

Dieting and non-dieting were measured by asking participants to respond to the questions: “I am currently dieting” and “I am currently eating healthily without dieting”, scored on a 7-point Likert scale ranging from 1 (definitely not) to 7 (definitely). We did not dichotomise this variable as a yes/no response format to accommodate people who may have made brief attempts at either and because of brevity not considered themselves dieting or non-dieting. Although the dieting definition has been used previously and is considered valid [53], the latter definition was adopted for this study and broadly represented non-dieting. Because dieting often involves external rules and regulations regarding eating, the opposite of this could be reliance on hunger and internal cues (i.e., intuitive eating; [15, 54, 55]). However, other non-dieting concepts and programs including competent eating [56], mindful eating [57], and the Appetite for Life Program [58] also fit under the umbrella of non-dieting approaches and so we adopted this broader non-dieting conceptualisation so long as it disavowed dieting methods and objectives. We assumed that non-dieting approaches would focus on eating healthily. This wider conceptualisation was consistent with the broad definition of self-reported dieting also defined by its objective (weight loss). Assessing non-dieting as the absence of dieting would not consider the focus on healthy eating emphasised by non-dieting advocates, while measuring non-dieting with a question solely concerning healthy eating would not explicitly convey an approach that disavowed dieting methods and objectives. Therefore, ‘eating healthy without dieting’ meant eating healthy without intentionally restricting caloric intake or eating certain types of foods for the purposes of losing weight and/or changing body shape. At Time 2, these measures correlated negatively (*r* = −.35, *p* [one-tailed] < .01), indicating that they were not considered complete opposites by participants and there was to some degree conceptual overlap (because diets would presumably encourage healthy eating too). To confirm that participants had interpreted these questions correctly, we asked participants at Time 2 four individual items to ascertain to what extent they had, in the past month: ‘changed their eating to lose weight’, ‘changed their eating to alter their body shape’, ‘restricted their calorie intake’ and ‘eaten healthily’. We used Spearman’s rho correlations (Table 1) because data appeared non-normal.

**Table 1:** Convergent and discriminant validity for dieting and non-dieting items (*n* = 450)

Items	Non-dieting dependent variable	Dieting dependent variable
Restricted calorie intake in the past month	-.24**	.71**
Changed eating to lose weight in the past month	-.26**	.74**
Changed eating to alter body shape in the past month	-.23**	.69**
Eaten healthily in the past month	.55**	.07

* *p* < .05 (1-tailed), ** *p* < .01 (1-tailed)

#### Independent variables

Most items were assessed using a 7-point Likert scale. The non-planning subscale measuring impulsivity used a 4-point Likert-type scale since the scale has been validated using this response format [59], while the attitudinal measures used a semantic-differential format (e.g., good-bad). Items with negatively worded endpoints were reverse-scored and recoded so the scale endpoints reflected positive or higher attributes of the construct. Items were averaged to create scale scores for cases with (at least) two thirds of the scale items completed. For the non-dieting items, the words ‘diet/dieting’ were substituted with the words ‘eat/eating healthily without dieting’.

### TPB constructs

Due to survey space constraints and because multiple-item intention measures often display Cronbach’s alphas > .90 [60], a single-item measure of intention was used for each behaviour. Consistent with previous studies [27], one item each assessed participants’ intention to diet or non-diet: “I intend to diet/eat healthily without dieting in the next month”, ranging from 1 *no*, *definitely not* to 7 *yes*, *definitely*. One item each assessed *past behaviour*: “I dieted in the past month” or “I ate healthily without dieting in the past month”. Based on a previous study [28], five items assessed *attitudes* towards dieting and non-dieting (10 items in total): “dieting/eating healthily without dieting in the next month would be 1 *harmful* to 7 *beneficial*; 1 *pleasant* to 7 *unpleasant*; 1 *foolish* to 7 *wise*; 1 *bad* to 7 *good*; 1 *ineffective* to 7 *effective*. We substituted unenjoyable-enjoyable for ineffective-effective because previous literature suggested that the effectiveness of a diet is a key attitudinal consideration [20]. Two injunctive items and one descriptive item assessed *subjective norm*: “Most people who are important to me think that I (should not/should) diet/eat healthily without dieting”; “It is expected of me that I will diet/eat healthily without dieting” and; “People who are important to me diet/eat healthily without dieting”, ranging from 1 *no*, *definitely not* to 7 *yes*, *definitely*. Two items drawn from a previous study [24] assessed *self*-*efficacy*: “How confident are you that you will be able to diet/eat healthily without dieting in the next month?” ranging from 1 *not confident at all* to 7 *very confident*, and “I believe I have the ability to diet/eat healthily without dieting in the next month” ranging from 1 *no*, *definitely not* to 7 *yes*, *definitely*.

*Self*-*identity* assessed how participants’ self-concept influenced their intentions to diet or non-diet. Based on findings of the prior qualitative study [18], we assessed dieting self-identity with three items: “I think of myself as someone who is concerned with my weight”, “I think of myself as a dieter”, and “I think of myself as an overweight person”, all ranging from 1 *no*, *definitely not* to 7 *yes*, *definitely*. For non-dieting, self-identity was assessed with two items: “I think of myself as a healthy eater”, and “I think of myself as someone who eats healthily without dieting”, both ranging from 1 *no*, *definitely not* to 7 *yes*, *definitely*. The non-dieting self-identity was a two-item scale because thinking of oneself as a “normal weight” person (in contrast to an overweight person) was not apparent in the previous qualitative study [18]. Although self-identity is usually operationalised using the same language for each question, the qualitative study suggested that this terminology clustered together, the internal consistency of the self-identity items supported this conceptualisation and previous studies applying self-identity in this context have used different language that could cluster together, such as healthy eating and pleasures of eating [24].

Weight control beliefs were measured with the Weight Control Beliefs Questionnaire [23], a 17-item, two-factor scale capturing two sets of beliefs: Beliefs that “you can and should control your weight” (BCWeight) and beliefs that “you should strive for a healthy lifestyle and accept the resulting weight” (BCLifestyle). BCWeight consists of beliefs that underlie weight control significant positive associations with disturbed eating, body dissatisfaction and poor self-esteem, whereas BCLifesyle assesses beliefs underlying non-dieting and has a strong protective relationship against these outcomes [23]. Laliberte et al. [23] have successfully discriminated between eating disordered and non-eating disordered individuals using the scales. These scales were validated in this study through examining correlations with theoretically related subscales in both dieting and non-dieting models. In this study, BCWeight was positively and significantly correlated with all measures assessing dieting in the dieting model. Conversely, BCLifestyle was significantly and positively related to all measures assessing non-dieting in the non-dieting model. No measure capturing attributions for diet adherence failure could be located in the literature so we measured this with a 1-item scale informed by the qualitative findings and invented for this study: “If I was not able to stick to a diet, it would be…” ranging from 1 *my fault*, 4 *my fault and the diet*’*s fault* to 7 *the diet*’*s fault*”. A two-item measure would have been preferable but we concluded that a midpoint proportioning the blame between the individual and the diet would adequately represent the construct. In support of this, this measure of dieting failure had a medium-to-large correlation with beliefs that you can and should control your weight at *r* = .47 (*p* < .01) and a medium-sized correlation with positive attitudes towards dieting *r* = .33 (*p* < .01), which we would expect given that blaming yourself rather than a diet would maintain positive attitudes towards dieting. Finally, we measured the personality/behavioural construct of impulsivity using the 5-item non-planning subscale of the Barratt Impulsiveness Scale – Version 11 [60].

In terms of moderators, self-reported height and weight were used to calculate body mass index (BMI) by dividing weight in kilograms by height in metres squared. BMI categories were calculated by recoding the continuous BMI variables into normal weight (BMI between 18.5 and 24.99), overweight (BMI between 25 and 29.99) and obese (BMI 30 or more) categories. Due to sample size considerations, data from overweight and obese participants were run together and compared to normal weight adults.

Please see the Additional file 1 for the full questionnaire used in this study.

### Statistical procedure

We used path modelling in IBM SPSS AMOS 21.0.0 [61] to test hypothesised relationships. We used the maximum likelihood (ML) estimation method to test the models and bootstrapped for robust standard errors and corrected test statistics when non-normality appeared. We evaluated overall model fit using the χ^2^ test and the “normed” χ^2^ statistic (χ^2^/df), because even minor deviations from a perfect model can result in a significant χ^2^ statistic with large sample sizes [62]. Values below 3.00 were deemed to indicate acceptable model fit [63]. We examined other absolute and incremental fit indices using Hu and Bentler’s [64] proposed cut-off values (Comparative Fit Index [CFI] > .95; Root Mean-Square Error of Approximation [RMSEA] < .06; Standardised Root Mean-square Residual [SRMR] < .08). We reported on two additional fit indices with cut-offs based on [65]: Adjusted Goodness-of-Fit index [AGFI] ≥ .95 and the Tucker Lewis Index (TLI) ≥ .95. Models were re-specified by assessing the critical ratio’s (t-values), standardised residual covariances, and modification indices suggested by AMOS. Re-specification was made only if the proposed relationships had a strong rationale based on theory, logic, or past research.

## Results

At Time 1, the majority of completing participants were from major cities (*n* = 640, 89%), followed by inner regional (*n* = 40, 5.6%), outer regional (*n* = 9, 1.3%) and remote areas (*n* = 6, 0.9%). Slightly more males (*n* = 375, 52.2%) than females (*n* = 344, 47.8%) participated, while the age of respondents varied from 18 to 76 years (*M* = 34.01 years, *SD* = 12.72 years). The majority of participants were employed full-time (*n* = 287, 39.9%), part-time or casual (*n* = 158, 22%), or were studying full-time (*n* = 203, 28.2%), while smaller numbers had home duties or were a carer (*n* = 17, 2.4%), were retired (*n* = 16, 2.2%) or were a part-time student (*n* = 12, 1.7%). Almost half of participants had BMIs in the healthy range (*n* = 346, 48.1%), while 32.3% (*n* = 232) were overweight and 19.6% (*n* = 141) were obese. Participants were generally well-educated, with 60.6% (*n* = 436) having completed a bachelor degree or higher, 13.2% (*n* = 95) completed a diploma or certificate and 23.6% (*n* = 170) having completed high school. At Time 2, 64% (*n* = 460) of participants reported their dieting and non-dieting behaviour over the last month. Participants reported dieting at a low level (*M* = 3.00, *SD* = 2.33) in the previous month while non-dieting was evident at a moderate level (*M* = 4.59, *SD* = 1.88). Table 2 below provides more Time 1 and Time 2 demographics. There were no statistically significant differences between the samples on any of the demographic variables from these two time periods.

**Table 2:** Social Demographics for Samples at Time 1 and Time 2 (1 month delay)

Characteristic	Time 1	Time 2	Difference (*p* value)
Age	*M* = 33.80	*M* = 35.10	.46
	*SD* = 12.83	*SD* = 12.81	
Gender			
- Male	389 (54.1%)	203 (43.8%)	.16
- Female	330 (45.9%)	261 (56.3%)	
Marital status			
- Married	246 (34.2%)	181 (39.0%)	.22
- De facto	125 (17.4%)	76 (16.4%)	
- Separated	13 (1.8%)	11 (2.4%)	
- Divorced	23 (3.2%)	13 (2.8%)	
- Widowed	4 (0.6%)	1 (0.2%)	
- Never married	297 (41.3%)	180 (38.8%)	
- Don’t know	6 (0.8%)	1 (0.2%)	
- Missing	5 (0.7%)	1 (0.2%)	
Education			
- Grade 10	2 (0.3%)	1 (0.2%)	.26
- High school	171 (23.8%)	92 (19.8%)	
- Bachelor degree or higher	430 (59.8%)	305 (65.7%)	
- Trade certificate (4 years duration)	8 (1.1%)	5 (1.1%)	
- Diploma of certificate	96 (13.4%)	53 (11.4%)	
- Don’t know	3 (0.4%)	1 (0.2%)	
- Prefer not to answer	1 (0.1%)	1 (0.2%)	
- Marine pilot	1 (0.1%)	0 (0%)	
- Missing	7 (1.0%)	6 (1.3%)	
Income			
- Less than $20,000	84 (11.7%)	44 (9.5%)	.23
- $20,001–$30,000	45 (6.3%)	32 (6.9%)	
- $30,001–$50,000	65 (9.0%)	35 (7.5%)	
- $50,001–$100,000	213 (29.6%)	149 (32.1%)	
- $100,001–$150,000	152 (21.1%)	105 (22.6%)	
- Over $150,000	101 (14.0%)	63 (13.6%)	
- Don’t know	31 (4.3%)	16 (3.4%)	
- Prefer not to answer	25 (3.5%)	17 (3.7%)	
- Missing	2 (0.4%)	3 (0.6%)	
BMI	*M* = 23.30, *SD* = 36.75	*M* = 30.33, *SD* = 64.78	.42
Required to diet			
- Yes	102 (14.2%)	63 (13.6%)	.16
- No	613 (85.3%)	398 (85.8%)	
- Missing	4 (0.5%)	3 (0.6%)	
Employment status			
- Employed, full-time	285 (39.6%)	194 (41.8%)	.26
- Employed part-time or casual	157 (21.8%)	106 (22.8%)	
- Home duties or carer	14 (1.9%)	12 (2.6%)	
- Unemployed	7 (1.0%)	3 (0.6%)	
- Full-time student	205 (28.5%)	114 (24.6%)	
- Part-time student	14 (1.9%)	10 (2.2%)	
- Retired	18 (2.5%)	13 (2.8%)	
- Permanently ill/unable to work	3 (0.4%)	2 (0.4%)	
- Prefer not to answer	5 (0.7%)	3 (0.6%)	
- Self-employed	6 (0.8%)	4 (0.9%)	
- Volunteer	1 (0.1%)	1 (0.1%)	
- Semi-retired	2 (0.3%)	2 (0.4%)	

Note: N for Time 1 = 719, N for Time 2 = 464. The continuous variables of Age and BMI were compared using *t*-tests, the categorical variables were compared using chi-square tests

**Fig. 1:**
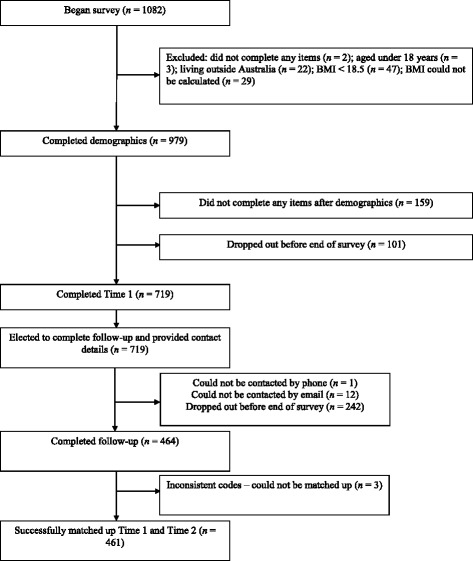
Flow chart of participants

Figure 1 shows the flow of participants through the baseline and follow-up questionnaires. We conducted preliminary analyses to identify any differences between Time 2 completers (*n* = 461) and non-completers (*n* = 258). A dummy variable was created with 0 = non-completers and 1 = completers and we ran chi-square analyses or t-tests comparing this variable to demographic factors. Chi-square tests indicated that Time 2 completers were significantly more likely to be female (*p* = < .001) or have completed a bachelor degree or higher (*p* < .001). No significant differences were observed between completers and non-completers on age, BMI, income, or area-level socio-economic status. Mann–Whitney U Tests and a one-way analysis of variance indicated that women were significantly more likely to diet than men (*p* < .001), and we observed a linear relationship between dieting and BMI status (*p* < .001).

Most predictor and criterion variables were positively and significantly correlated (see Tables 3 and 4 below). In the dieting model, non-planning was unrelated to dieting self-efficacy, BCWeight, past behaviour, intention, and behaviour. Non-planning was still retained in the multivariate model since we hypothesised it may be more important for different subgroups who were more inclined to diet (females, overweight and obese adults). For the non-dieting model, non-planning (reverse-scored) was positively correlated with all variables, except for dieting failure attribution (DFA). All other variables correlated in the expected direction. In both models, all variables correlated more strongly with intentions than behaviour, except for planning in the dieting model, which correlated higher with behaviour. While the strongest correlate of intention and behaviour in the dieting model was past behaviour, the correlations of self-identity and self-efficacy to non-dieting behaviour were equivalent and stronger than the relationship between past behaviour and behaviour, respectively.

**Table 3:** Means, Standard Deviations, Spearman’s Rho Bivariate Correlations, and Reliability (Cronbach’s Alpha) for Predictor and Outcome Dieting Variables (*N* = 456)

Variable	*M* (*SD*)	*1*	*2*	*3*	*4*	*5*	*6*	*7*	*8*	*9*
1. Attitude	4.19 (1.59)	(.91)								
2. Subjective norm	3.21 (1.53)	.49**	(.68)							
3. Self-efficacy	4.82 (1.77)	.47**	.14**	(.90)						
4. Self-identity	3.55 (1.61)	.48**	.55**	.08*	(.70)					
5. Non-planning	9.13 (2.82)	.13**	.13**	.00	.09*	(.73)				
6. DFA	5.26 (1.68)	.33**	.14**	.26**	.16**	.10*	-			
7. BCWeight	43.67 (8.50)	.36**	.08*	.30**	.14**	-.04	.47**	(.86)		
8. Past behaviour	3.03 (2.42)	.56**	.37**	.35**	.52**	.01	.16**	.19**	-	
9. Intention	3.37 (2.40)	.71**	.50**	.35**	.62**	.08	.22**	.25**	.78**	-
10. Behaviour	2.99 (2.32)	.54**	.36**	.30**	.46**	.05	.14**	.21**	.69**	.67**

Note. Non-planning scores ranged from 5 to 20. BCWeight scores ranged from 14 to 56, *DFA* dieting failure attributions (diet’s fault 1, my fault = 7), * *p* < .05, ** *p* < .01

**Table 4:** Means, Standard Deviations, Spearman’s Rho Bivariate Correlations, and Reliability (Cronbach’s Alpha) for Predictor and Outcome Non-dieting Variables (*N* = 444)

Variable	*M* (*SD*)	*1*	*2*	*3*	*4*	*5*	*6*	*7*	*8*	*9*
1. Attitude	5.95 (1.16)	(.87)								
2. Subjective norm	5.18 (1.22)	.49**	(.64)							
3. Self-efficacy	5.32 (1.73)	.60**	.40**	(.88)						
4. Self-identity	4.91 (1.62)	.49**	.42**	.73**	(.83)					
5. Planning	15.80 (2.83)	.20**	.25**	.19**	.29**	(.73)				
6. DFA	2.75 (1.69)	.17**	.15**	.13**	.19**	.07	-			
7. BCLifestyle	41.99 (11.83)	.51**	.40**	.62**	.60**	.16**	.22**	(.92)		
8. Past behaviour	5.04 (1.89)	.50**	.36**	.64**	.72**	.22**	17**	.48**	-	
9. Intention	5.52 (1.80)	.56**	.42**	.60**	.62**	.20**	23**	.49**	.70**	-
10. Behaviour	4.63 (1.86)	.50**	.36**	.57**	.55**	.21**	.10*	.44**	.55**	.54**

Note. Non-planning recoded so higher scores indicate planning. BCLifestyle scores ranged from 9 to 63, *DFA* dieting failure attributions (my fault = 1, diet’s fault = 7), * *p* < .05, ** *p* < .01

### Dieting model

We imputed 28 (0.2%) data points using Expectation Maximisation (EM) estimation for participants who had completed the Time 2 dieting dependent variable but missed items in the Time 1 questionnaire. Little’s Missing Completely at Random (MCAR) test indicated that the data were missing at random (MAR) χ^2^ (173, *N* = 460) = 157.12, *p* = .80. We ran a multiple regression with the hypothesised variables to obtain Mahalanobis distance figures to examine for multivariate outliers. Using a chi-square table with 9° of freedom, the critical cut point of an alpha level of .001 was 27.79. Four cases scoring above this cut point were identified and removed from the dataset.

We assessed multivariate non-normality using Mardia’s normalized estimate of multivariate kurtosis [66, 67], using a criterion of greater than 5 as evidence of non-normality [68]. The initial run of both hypothesised models suggested multivariate non-normality, so we made post-hoc adjustments to the chi-squared statistic and the standard error estimates to account for inflated chi-squared tests of model fit and underestimated standard errors in non-normal data. To adjust the chi-square statistic, we used the Bollen-Stine bootstrap *p* for overall correction to adjust the chi-square statistic and, in a separate run, performed 500 bootstraps [61] to correct standard errors for non-normality. No descriptive statistics indicated the presence of linearity/multicollinearity in the sample and no error warnings indicating their presence were received during runs in AMOS.

The hypothesised dieting model allowed all independent variables to predict intention to assess their relative contribution to explaining the variance in intention. Intention, self-efficacy, and self-identity predicted behaviour directly. Self-efficacy may predict behaviour directly when individuals realistically estimate their control over behaviour. The self-identity and behaviour relationship is based on previous research hypothesising a direct link in certain situations [45]. Non-planning predicted behaviour directly based on previous studies [38, 39]. Past behaviour predicted the standard TPB constructs (including intention and behaviour) and all additional influences, based on our previous findings. The TPB predictor variables and self-identity were allowed to co-vary with each other based on the theory. We expected that BCWeight would covary with all TPB variables, including self-identity, and so these covariance terms were added prior to the initial run. We also expected that internal attributions for dieting failure (DFA) would relate to positive attitudes about dieting (since diets themselves are not implicated in failure), and so a covariance term was added between these variables.

The initial model indicated multivariate non-normality, with Mardia’s test suggesting a critical ratio of 5.861. A Bollen-Stine bootstrap with each model was subsequently run to adjust chi-square significance values for the presence of non-normality. The initial dieting model was almost a good fit, χ^2^ (13) = 64.32, *p* < .01, Bollen-Stine Bootstrap *p* = .01, normed χ^2^ = 4.95, AGFI = .89, TLI = .91, CFI = .97, RMSEA = .09, SRMR = .05. Modification indices and standardised residual covariances suggested improving the model by covarying DFA with (1) self-identity, (2) self-efficacy, and (3) subjective norm. Although a positive relationship between DFA and dieting self-efficacy seems counterintuitive, it is possible to hold these two beliefs simultaneously, as individuals attributing their diet failure to themselves but as an adaptive strategy still endorse items that they believe in and have confidence in their ability to diet. The moderate correlation between the two items supported this argument.

**Fig. 2:**
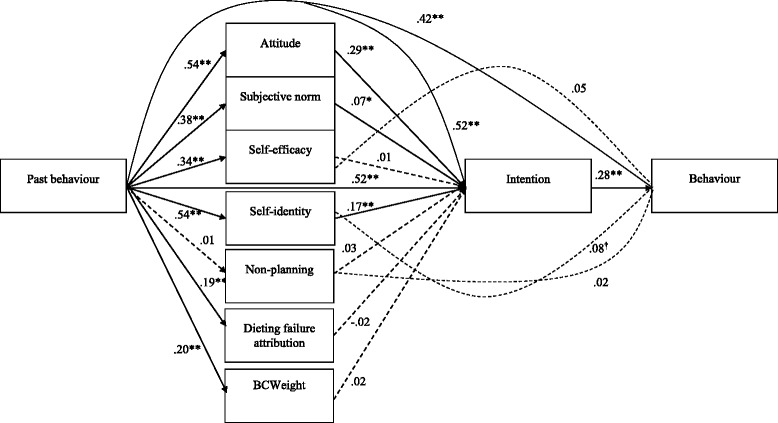
Final path model depicting predictors of dieting intentions and behaviour (*N* = 456). Note. This figure does not show covariance paths. ^†^ *p* < .10, * *p* < .05, ** *p* < .01, *** *p* < .001. Solid lines denote significant paths and dotted lined denote non-significant paths

The final model fitted the data well, χ^2^ (11) = 25.30, *p* = .01, Bollen-Stine Bootstrap *p* = .01, normed χ^2^ = 2.3, AGFI = .95, TLI = .97, CFI = .99, RMSEA = .05, SRMR = .03. The AGFI index was slightly below the desired level of > 0.95 although any further changes were considered illogical and not parsimonious. In the final model, past behaviour, attitude, subjective norms, and self-identity significantly predicted dieting intentions. Intention and past behaviour significantly predicted dieting behaviour, while self-identity and self-efficacy approached significance. The full model explained 74.8% of the variance in dieting intention and 52.9% of the variance in Time 2 dieting behaviour. Figure 2 indicates the standardised regression weights of each variable and their associated significance values based on the bias-corrected confidence intervals (set at 95%). We report these because of non-normality, the discrepancies noted between standard errors and bootstrapped standard errors and because they are considered to yield more accurate values than percentile method intervals [69].

Because predictors of dieting might vary by gender and BMI, we re-ran the final model separately to compare: (1) males (*n* = 199) and females (*n* = 257), then (2) normal weight (*n* = 250) vs overweight and obese (*n* = 206). For all subgroup analyses, we based sample size adequacy on recommendations from Weston and Gore [70], who suggest that more than 200 is considered adequate. For males, subjective norms did not predict intention (β = .06, *p* = .24), DFA predicted dieting intention, albeit in an unexpected direction (β = −.09, *p* = .04), and BCWeight predicted dieting intention (β = .08, *p* = .04). For females, subjective norms did not significantly predict intentions (β = .08, *p* = .08), while DFA (β = .05, *p* = .09) and non-planning (β = .05, *p* = .09) almost predicted dieting intentions. Self-identity predicted dieting behaviour directly (β = .18, *p* = .01) but intentions no longer predicted behaviour (β = .14, *p* = .12). Subjective norms remained a significant predictor of dieting intentions for normal weight adults (β = .06, *p* = .049) but all other findings were the same as the total model. For overweight and obese adults, subjective norms (β = .11, *p* = .06) and self-efficacy (β = .08, *p* = .09) did not significantly predict dieting intentions. Intentions no longer predicted behaviour (β = .208, *p* = .084). No other notable differences from the total model emerged.

In addition to testing the above theoretical constructs, we also performed a confirmatory factor analysis (CFA) in order to test the consistency of the constructs with the observed data. The original model included all of the items that were proposed to constitute the latent variables described in the method section. This model had a less than adequate fit, χ^2^ (284) = 1116.992, *p* < .001, Bollen-Stine Bootstrap *p* = .002, normed χ^2^ = 3.933, AGFI = .794, TLI = .842, CFI = .862, RMSEA = .0831, SRMR = .0794. The following items were then deleted from the CFA due to having estimates of less than .70: non-planning (items one, two and three), attitudes (item two), subjective norm (item three) and BCWeight (items one, four, six, seven and eight). The CFA was then repeated and the model fitted the data well, χ^2^ (75) = 217.857, *p* < .001, Bollen-Stine Bootstrap *p* = .002, normed χ^2^ = 2.905, AGFI = .908, TLI = .950, CFI = .965, RMSEA = .065, SRMR = .0498 .

The observed and latent variables that were included in the final CFA were then added to the original path analysis model for non-dieting behaviour described above and tested using the same methodology. This model had a poor fit with the data, χ^2^ (127) = 444.2, *p* < .001, Bollen-Stine Bootstrap *p* = .002, normed χ^2^ = 3.526, AGFI = .863, TLI = .919, CFI = .940, RMSEA = .075, SRMR = .0779. The model though did account for 74.7% of the variance in intention and 52.7% of the variance in behaviour.

Due to the relatively poor fit of the model, the model was further refined by deleting all non-significant associations. This involved deleting BCWeight-Intention, Self-Efficacy-Intention, Self-Identity-Intention, Subjective Norm-Intention, Non-Planning-Intention, Attribution of Failure-Intention, Past Behaviour-Non-Planning, Non-Planning-Time 2 Behaviour and Past Behaviour-Time 2 Behaviour, Self-Identity- Time 2 Behaviour, and Self-Efficacy-Time 2 Behaviour.

The final model had a worse fit and so was uninterpretable, χ^2^ (105) =791.64, *p* < .001, Bollen-Stine Bootstrap *p* = .002, normed χ^2^ = 7.539, AGFI = .770, TLI = .826, CFI = .866, RMSEA = .120, SRMR = .100. Given this poor fit we chose to stay with the final model based upon the standard measurement of the constructs as described above and depicted in Fig. 2 as best representation of the relationships found in this study.

### Non-dieting model

For the non-dieting model, we imputed 31 data points (0.23% of all values) using EM estimation on participants who completed the Time 2 dependent variable but had missed Time 1 items. Little’s MCAR test indicated that the data were MAR χ^2^ (257, *N* = 451) = 281.81, *p* = .14. To identify multivariate outliers, we ran a multiple regression with the hypothesised variables to obtain Mahalanobis distance figures. Using a chi-square table with 9° of freedom, the critical cutpoint of an alpha level of .001 was 27.79. Seven cases scored above this cut point and were removed.

The initial hypothesised model was identical to the dieting model, except that DFA and non-planning were recoded so higher scores indicated that the diet was responsible for failure (external attribution) and so that non-planning could be covaried with other variables based on positive hypothesised relationships between planning and non-dieting. The hypothesised non-dieting model contained the same predictors with the exception of different items for self-identity and the inclusion of BCLifestyle instead of BCWeight.

The initial model suggested multivariate nonnormality, with Mardia’s test indicating a critical ratio of 12.52. We ran a Bollen-Stine bootstrap with each model run to adjust the chi-square significance value for non-normality. The initial non-dieting model was almost a good fit to the data, χ^2^ (13) = 50.17, *p* < .01, Bollen-Stine Bootstrap *p* = .01, normed χ^2^ = 3.86, AGFI = .91, TLI = .93, CFI = .98, RMSEA = .08, SRMR = .04. Modification indices and standardised residual covariances suggested improving the model by covarying DFA with self-identity and subjective norm. Self-identity as a healthy person and subjective norms were covaried with planning as suggested by the standardised residual covariances.

**Fig. 3:**
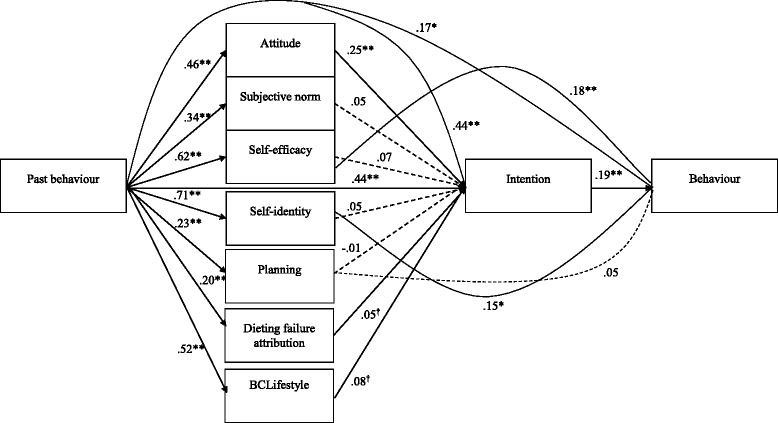
Final path model depicting predictors of non-dieting intentions and behaviour (N =, 444). Note. This figure does not show covariance paths. ^†^ *p* < .10, * *p* < .05, ** *p* < .01, *** *p* < .001. Solid lines denote significant paths and dotted lined denote non-significant paths

The final model fitted the data well, χ^2^ (9) = 15.65, *p* = .08, Bollen-Stine Bootstrap *p* = .09, normed χ^2^ = 1.74, AGFI = .96, TLI = .98, CFI = .99, RMSEA = .04, SRMR = .02. Past behaviour and attitude predicted non-dieting intentions while BCLifestyle and DFA almost predicted non-dieting intentions. Past behaviour, self-identity and self-efficacy directly predicted non-dieting behaviour. The model explained 58.2% of variance in intention and 37.5% of variance in behaviour (see Fig. 3).

In addition to testing the above theoretical constructs, we also performed a confirmatory factor analysis (CFA) to test the consistency of the constructs with the observed data. The original CFA model included all the items that were proposed to constitute the latent variables described in the method section. This model had a less than adequate fit, χ^2^ (286) = 957.60, *p* < .001, Bollen-Stine Bootstrap *p* = .002, normed χ^2^ = 3.348, AGFI = .822, TLI = .892, CFI = .905, RMSEA = .072, SRMR = .0705. The following items were then deleted from the CFA due to having estimates lower than .70: non-planning (items one, two and three), attitudes (item two), subjective norm (item three) and BCLifestyle (items four, five, six and seven). The CFA was then repeated and the model fit was improved, χ^2^ (158) = 388.875, *p* < .001, Bollen-Stine Bootstrap *p* = .002, normed χ^2^ = 2.461, AGFI = .892, TLI = .952, CFI = .964, RMSEA = .057, SRMR = .0612.

The observed and latent variables that were included in the final CFA were then added to the original path analysis model for non-dieting behaviour described above and tested using the same methodology. This model fitted the data relatively well, χ^2^ (158) = 388.875, *p* < .001, Bollen-Stine Bootstrap *p* = .002, normed χ^2^ = 2.461, AGFI = .892, TLI = .952, CFI = .964, RMSEA = .057, SRMR = .0612. The model accounted for 58% of the variance in intention and 39.2% of the variance in behaviour.

While the fit of the model was adequate and the amount of variance explained by the model was similar to the theoretical model, this model was further refined by deleting all non-significant associations. This involved deleting self-identity –intention, BCLifestyle-Intention, Social Norm-Intention, Self-Efficacy-Intention, Non-Planning-Intention, Attribution of Failure-Intention, Non-Planning-Time 2 Behaviour and Past Behaviour-Time 2 Behaviour. The final model had a similar fit, χ^2^ (164) =400.20, *p* < .001, Bollen-Stine Bootstrap *p* = .002, normed χ^2^ = 2.439, AGFI = .893, TLI = .953, CFI = .963, RMSEA = .057, SRMR = .0627. The new model however did not significantly increase the amount of variance accounted for in intention (57.6%) or in behaviour (38.6%). In this model, past behaviour (β = .301, *p* = .001), attitudes (β = .287, *p* < .001), self-identity (β = .224, *p* = .003)) and BCLifestyle (β ==.120, *p* = .043) were all independently associated with non-diet intention, while only non-diet intention (β = .188, *p* < .001), self-identity (β = .328, *p* < .001) and self-efficacy (β = .182, *p* = .018) predicted non-diet behaviour.

This data-driven model did not significantly improve the model fit nor the amount of variance explained in intention and behaviour compared with the model that used the theoretical constructs. Therefore, to allow easier comparison of our findings to findings from other past and future studies that used the same measures of theoretical constructs, we retained the final model depicted in Fig. 3 as the best representation of the relationships found in this study.

Consistent with the dieting model, we re-ran the final model separately to compare: (1) males (*n* = 198) and females (*n* = 246), and (2) normal weight (*n* = 242) compared to overweight and obese individuals (*n* = 202). For males, self-identity no longer predicted behaviour (β = .08, *p* = .45) but all other predictors were similar. For females, subjective norms (β = .09, *p* = .03), BClifestyle (β = .15, *p* = .02), self-efficacy (β = .04, *p* = .04) and attributing the failure of diets to the diet itself (β = .07, *p* = .046) all predicted non-dieting intention. Surprisingly, past behaviour (β = .17, *p* = .05) and intention (β = .17, *p* = .08) only approached significance in predicting non-dieting behaviour, due to the wider confidence intervals for the standardised estimates. While self-efficacy (β = .09, *p* = .31) did not predict behaviour either, self-identity (β = .21, *p* = .02) retained its influence. For normal weight adults, DFA almost predicted non-dieting intentions (β = .06, *p* = .08). Self-efficacy (β = .25, *p* = .01) and intention (β = .26, *p* = .01) remained as predictors of non-dieting behaviour but self-identity did not (β = .07, *p* = .45). For overweight and obese individuals, attributions for dieting failure were not relevant to the prediction of intentions (β = .06, *p* = .31) but BCLifestyle beliefs were (β = .15, *p* = .02). The only predictor of non-dieting behaviour was planning (β = .14, *p* = .02), while intention (β = .17, *p* = .07) and self-identity (β = .19, *p* = .09) were quite close to significance.

## Discussion

The present findings suggest that determinants of dieting and non-dieting attempts include attitudes towards each behaviour, subjective norms (only for dieting), past behaviour, self-identity, and self-efficacy (non-dieting only). Notably, self-identity and self-efficacy predicted behaviour directly in the non-dieting model. The hypotheses that some of these determinants would be opposite ends of a continuum, for items that were reverse-scored, were not supported. Specifically, attributions for dieting failure were not significant in either model for the total sample, and neither was non-planning. Weight control beliefs also did not differ in their impact (both non-significant) on dieting and non-dieting intentions in the total sample analyses.

Consistent with our dieting model hypotheses, attitudes, subjective norm, self-identity, and past behaviour all predicted dieting intentions. Inconsistent with hypotheses though, there was no effect for the other variables hypothesised to be of relevance in the total sample. Self-efficacy and self-identity nearly predicted dieting behaviour, while non-planning did not. Despite this, the variance explained in intentions (74.8%) and behaviour (52.9%) was high and is comparable to the variance of intentions (77%) and behaviour (46%) explained in a previous study [27] that used a slightly different definition of dieting (including both weight loss and weight loss maintenance). The variance explained compares favourably with the predictive power of standard TPB variables reported in meta-analyses [29]. Where an average of 21.2% of variance in dietary behaviour is explained.

Our findings suggest that dieting intentions are predicted most strongly by favourable attitudes towards dieting (attitudes); expectations of others that you should diet (injunctive norms) or the actual dieting behaviours of others (descriptive norms); and seeing yourself as a dieter, overweight person and weight concerned person (self-identity). These dieting intentions subsequently predict the likelihood of attempting to diet. The strong effect of attitudes on behaviour in this context is consistent with previous studies [27] that found attitudes to be the strongest predictor of dieting behaviour. In contrast though, injunctive norms were not associated with dieting intentions in a female university sample [28]. Earlier applications of the TPB to dieting have found an effect for descriptive norms in a high school sample [71]. Overall, we attribute the lack of support for dieting self-efficacy to the TPB’s limitations (see [72]), and our findings are consistent with previous studies [24] that have found no effect of self-efficacy on low-fat dietary consumption. That self-identity is a stronger predictor of intentions than subjective norms and self-efficacy is consistent with [35] and suggests that it is assists in understanding why adults form dieting intentions.

Post-hoc analyses of subgroups suggested that, in this sample at least, beliefs that you can and should control your weight are more relevant to predicting dieting intentions in males than females. Given that these beliefs are associated with body dissatisfaction, drive for thinness, and disordered eating in females [23], it would be important to see if these same associations exist in men. If this is not the case, health promoters who wish to encourage calorie-restricted dieting approaches in men could promote beliefs that weight can and should be controlled. The findings for DFA were surprising, with men who thought they were responsible for failing to stick to a diet less inclined to intend to diet, while the opposite effect was almost observed for women. Perhaps these findings speak to different levels of engagement with dieting, since women are more likely to engage in weight control efforts than men [16, 73]. The finding that self-identity directly predicted attempts to diet directly for women suggests that this identity may be influential in decision-making through directing individuals to act in a way (diet) consistent with their identity. The finding that intentions did not predict behaviour for women would seem to support this assertion that there are other post-intentional influences on behaviour.

The absence of the intention-behaviour relationship was also observed in overweight and obese individuals. Although almost significant, the wide confidence intervals for the intention beta weight suggests that there is considerable heterogeneity for the translation of intentions into behaviour for this subgroup, which could conceivably be influenced by post-intentional factors including motivation, and the strength of commitment to the intention [74]. The finding that subjective norms only predicted dieting intentions for normal weight individuals is surprising, and indicates that other people are more influential for this group because motivation to diet due to body dissatisfaction is not as high as it may be in other groups (e.g., women and overweight/obese), who may instead intend to diet based on appearance, health, or mood [75].

In the non-dieting model, past behaviour and attitude predicted non-dieting intentions, while BCLifestyle and DFA almost predicted non-dieting intentions. Past behaviour, self-identity, and self-efficacy had direct effects on non-dieting behaviour. The non-dieting model explained 56% of the variance in intention and 37% of the variance in behaviour, which also compares favourably to the variance explained (23.1%) in studies concerning healthy dietary behaviour [29].

The findings that self-identity and self-efficacy influence non-dieting behaviour directly and almost as strongly as intentions suggest that both constructs may be important in the post-intentional phase. While, as expected, attitude influenced intentions, belief in one’s ability to eat healthily without dieting may promote efforts to persist with actual attempts at non-dieting behaviour. We speculate that healthy eating plans are not as visible or widely disseminated as dieting plans, which would mean that self-efficacy is more important in predicting behaviour because it is more critical to the development of plans which will facilitate behavioural initiation [76]. For self-identity, the findings are consistent with our qualitative study [18], which suggest that thinking of oneself as a healthy person tends to inform momentary behaviour (e.g., I am someone who eats healthily without dieting; what would I do in this situation?).

Our subgroup analyses found that self-identity significantly predicted non-dieting behaviour only in women, suggesting it was not a salient identity to men. Because women diet more often [16, 73], it may be that women who don’t diet more frequently invoke this self-identity to reinforce to themselves the desirability of non-dieting behaviour. For females, BCLifestyle predicted non-dieting intentions, as did external attributions towards dieting failure (the diet’s fault) and non-dieting self-efficacy. Surprisingly though, intentions and past behaviour did not predict actual non-dieting attempts, suggesting that behaviour may be influenced by more momentary processes such as self-identity. It also seemed that external attributions for dieting failure were more relevant to the prediction of non-dieting intentions for normal weight adults than they were for overweight and obese individuals, who might continue intending to diet due to their desire to lose weight [20]. For overweight and obese individuals, BCLifestyle predicted intentions. In other words, beliefs that you should accept your weight and work towards a healthy lifestyle increase the likelihood of overweight and obese individuals forming intentions to eat healthily without dieting. The relationship observed between non-planning impulsivity and attempts at eating healthily without dieting can be related to the two studies previously adding impulsivity to the TPB [39, 40], which both found that impulsivity significantly contributed to the prediction of high-calorie snack avoidance over and above the TPB constructs. Our findings suggest, conversely, that scoring lower on the impulsivity sub trait non-planning predicted non-dieting behaviour for overweight and obese individuals. This may be because the perceived benefits of eating healthily are probably less tangible (i.e., no immediate weight loss or change in body shape) and any perceived benefits will take longer to observe, therefore suiting future-oriented people who make long-term plans. Further investigation should ascertain whether attempts to undertake other health-promoting behaviours that have no immediate reward are associated with low impulsivity and examine mechanisms for these findings [77].

### Comparisons between models

In the total sample for each model, the findings were not dramatically different, except that subjective norms was related to dieting intentions but not non-dieting intentions. BCLifestyle was closer to predicting non-dieting intentions than BCWeight for dieting intentions. Hypotheses for direct prediction of behaviour were supported more strongly in the dieting model, where self-identity and self-efficacy predicted behaviour directly, indicating that this post-intentional phase is more important overall for the non-dieting model. This statement however, tends to obscure the widely varying relationships between intentions and behaviour that were observed in population subgroups. For instance, the intention-behaviour relationship was non-significant, albeit slightly, for women and overweight and obese individuals in both models, which may suggest that there are more influential post-intentional factors in these groups.

In terms of self-identity, both models provided support for the inclusion of a broad, multi-faceted identity from our qualitative study. For dieters, this identity consisted of being someone who diets, is concerned about their weight, and thinks of themselves as an overweight person, the non-dieting self-identity that predicted intentions concerned thinking of oneself as a healthy eater and someone who eats healthily without dieting. These findings support the application of identity theory [31] in the context of both behaviours, and are consistent with the wealth of qualitative literature that has consistently identified self-identity as an important variable [49, 78].

### Strengths and limitations

All data were collected by self-report, and there is evidence that the TPB is weaker at predicting behaviour that is relatively more objective than self-perceptions [24]. This is a limitation of both the data we collected but also the TPB. However, self-perceptions that influence behaviour are important, since clinicians and health promoters who have the objective of promoting each approach must understand, in the words of individuals, why they are motivated to adopt each approach. In either case, further research is necessary with objective measures of behaviour and longer follow-up periods.

Although subjective norm successfully predicted dieting, several different norms could conceivably influence dieting and non-dieting intentions. Group norms [79] might also predict dieting intention and behaviour, given that dieting is often undertaken in groups [20]. Moral judgments are attached to body shape and weight loss [19, 80], so personal moral norm [81] might contribute to dieting intentions. Since subjective norm was only significant in the dieting model, differences in autonomy might reflect these influences, with individuals low in autonomous motivation more inclined to diet and individuals high in autonomy inclined to eat healthily without dieting.

The measure of impulsivity used in this study is generic in nature, and a measure specific to health-related domains could have more predictive power if it corresponds to the behavioural context. Our impulsivity measure also differs from the scales used in previous studies [38, 39] and so efforts should be made to ascertain whether other measures of impulsivity are related to self-reported dieting and non-dieting behaviours. Although our measure of DFA did predict intentions and behaviour, a multi-item measure displaying internal consistency would be desirable for future studies.

Despite these limitations, we made efforts to recruit a community sample not primarily composed of undergraduate students. Males were well-represented, which is important because they are underrepresented in research concerning lifestyle change and weight loss [81]. The design assists in understanding why individuals adopt either approach in a naturalistic setting, which is crucial, given that some individuals will make attempts to diet or non-diet without assistance from health professionals [72, 82]. The use of a theory-based decision model supplemented with non-reflective (impulsivity) and domain-specific (weight control beliefs) measures explained a considerable proportion of the variance in dieting and non-dieting attempts, and provided further support for the application and integration of these concepts in with adults, overweight and obese individuals and males. Both models provided support for application of constructs from the TPB, identity theory, and locus of control framework, albeit in certain subgroups.

### Implications for practice and research

While attitudes are ever important, health promoters and clinicians should keep in mind that they are, in effect, attempting to develop new identities for individuals during behaviour change [30]. Interventionists can bridge the intention-behaviour gap with plans and consider fundamental weight control beliefs that individuals have when implementing lifestyle change programs since our findings suggest that these readily influence dieting and non-dieting intentions for certain subgroups. Interventions to increase dieting behaviour among suitable individuals should focus on increasing positive attitudes, subjective norms, and self-identity as a dieter. Interventions to increase non-dieting behaviour should focus on increasing positive attitudes towards non-dieting, negotiate the identity change that occurs as one moves to non-dieting from dieting, and focus on increasing messaging that improves self-efficacy to non-diet and self-identity as a non-dieter, both of which appear to be important direct predictors of non-dieting behaviour. While clinicians may be able to easily recognise clients who are unsuitable for either dieting or non-dieting approaches, future research in this area could further identify specific population subgroups who are suitable and unsuitable for dieting and non-dieting approaches, or may require a combination of the two approaches. Such research would enable more specific prediction models that built on the models tested here.

## Conclusions

This study suggests that dieting and non-dieting behaviours have different determinants that differ according to the subgroup under investigation. While attitudes are important across both behaviours, subjective norms appear more influential in the context of dieting. Self-efficacy is more influential to non-dieting behaviour than it is to dieting behaviour, while self-identity operated on both intentions and behaviour. These findings add further support for the utility of the TPB and identity theory in this area, while also supporting recent suggestions that impulsivity (or the absence of it) may play a role in predicting behaviour directly. Some aspects of the TPB, such as subjective norms and self-efficacy, were only partially supported. Beliefs that you should control your lifestyle and accept your weight were predictive of non-dieting intentions for females and overweight/obese individuals, while an effect for beliefs that you can and should control your weight on dieting intentions was only observed in men. Clinicians wishing to encourage dieting and non-dieting approaches should seek to promote these beliefs in their clients.

## Additional file

Additional file 1: Predictors of dieting and non-dieting approaches among adults living in Australia. This file includes the questionnaires used in the study examining the psychological predictors of dieting and non-dieting approaches among adults living in Australia. (DOCX 866 kb)

## Abbreviations

AGFI: Adjusted Goodness-of-Fit index
AMOS: Analysis of moment structures
BCLifestyle: Beliefs that you should strive for a healthy lifestyle and accept the resulting weight
BCWeight: Beliefs that you can and should control your weight
BMI: Body mass index
CFI: Comparative fit index
DFA: Dieting failure attribution
EM: Expectation Maximisation (EM)
IBM: International business machines corporation
MAR: Missing at random
MCAR: Missing Completely at Random
ML: Maximum likelihood
RMSEA: Root mean-square error of approximation
SPSS: Statistical package for the social sciences
SRMR: Standardised root mean-square residual
TLI: Tucker lewis index
TPB: Theory of planned behavior
