# Supplementary material for: Predictors of dieting and non-dieting approaches among adults living in Australia
Source: BMC Public Health. 2017 Feb 20;17:214. doi: 10.1186/s12889-017-4131-0 (PMC5319048; doi:10.1186/s12889-017-4131-0)
Supplement: Additional file 1: — Predictors of dieting and non-dieting approaches among adults living in Australia. This file includes the questionnaires used in the study examining the psychological predictors of dieting and non-dieting approaches among adults living in Australia. (DOCX 866 kb) [file 12889_2017_4131_MOESM1_ESM.docx]

Contents

[Study 3 Time 1 questionnaire 1](#_Toc469999619)

[Study 3 Time 2 questionnaire 16](#_Toc469999620)

Study 3 Time 1 questionnaire

**Beliefs about dieting, healthy eating, and weight control**
QUT Ethics Approval Number: 1300000130

RESEARCH TEAM
Principal Researcher: Stuart Leske, PhD student, Queensland University of Technology (QUT)
Associate Researchers: Dr Esben Strodl and Dr Xiang-Yu Hou, QUT; Ms Catherine Harper, Queensland Health

DESCRIPTION
This project is being undertaken as part of a PhD for Stuart Leske. The purpose of this project is for the researchers to better understand your beliefs about dieting, healthy eating, and weight control. You are invited to participate in this project because you are living in Australia and aged 18 years and over.

PARTICIPATION
Your participation will involve completing two anonymous surveys. The first survey contains 149 items with Likert scale answers (strongly disagree – strongly agree) that will take approximately 30 minutes of your time. Questions will include: “I trust my body to tell me what to eat”, “I believe I should control my weight”, and “I think of myself as a healthy eater”. This will be followed by a second survey 4 weeks later consisting of 7 items which will take approximately 5 minutes to answer.

Your participation in this project is entirely voluntary. If you agree to participate you do not have to complete any question(s) you are uncomfortable answering. Your decision to participate or not participate will in no way impact upon your current or future relationship with QUT (for example your grades). If you do agree to participate you can withdraw from the project at any time without comment or penalty. Any personal information already obtained from you for the purpose of entering you in the prize draw will be destroyed.

EXPECTED BENEFITS
It is expected that this project will not benefit you directly. However, the findings may benefit the wider Australian community by providing a better understanding of dieting, healthy eating, and weight control in adult Australians. This knowledge will allow health professionals to provide appropriate advice on dieting, healthy eating, and weight control to the Australian population.

To recognise your contribution if you choose to participate, the research team is offering the chance to win 1 of 3 iPad minis to the value of $369 each. You will be entered into this prize draw each time you complete a survey (2 times). First year psychology students in the unit PYB102 can instead choose to earn 0.5 of a mark (course credit) for completing the survey.

RISKS
There are no risks beyond normal day-to-day living associated with your participation in this project.

PRIVACY AND CONFIDENTIALITY
All comments and responses are anonymous and will be treated confidentially unless required by law. Your contact details collected for the prize draw will be stored separately from your data and only used to contact you if you win one of the prizes. Any data collected as part of this project will be stored securely as per QUT’s Management of research data policy. Non-identifiable data collected in this project may be used as comparative data in future projects and/or stored with the Australian National Data Service or similar to be used for secondary analysis in future projects.

CONSENT TO PARTICIPATE
Submitting the completed online survey is accepted as your consent to participate in this project. If you elect to have a survey mailed out to you, the return of the completed survey is accepted as your consent to participate in this project.

QUESTIONS/FURTHER INFORMATION ABOUT THE PROJECT
If you have any questions or require further information, please contact one of the research team members below.

Stuart Leske, School of Psychology and Counselling, Faculty of Health, phone: (07) 3138 4685, email: s.leske@qut.edu.au
Dr Esben Strodl, School of Psychology and Counselling, Faculty of Health, phone: (07) 3138 8415, email: e.strodl@qut.edu.au
Dr Xiang-Yu Hou, School of Public Health and Social Work, Faculty of Health, phone: (07) 3138 9240, email: x.hou@qut.edu.au
Ms Catherine Harper, Director, Population Epidemiology Unit, Division of the Chief Health Officer, email: Catherine_Harper@health.qld.gov.au

CONCERNS / COMPLAINTS REGARDING THE CONDUCT OF THE PROJECT
QUT is committed to research integrity and the ethical conduct of research projects. However, if you do have any concerns or complaints about the ethical conduct of the project you may contact the QUT Research Ethics Unit on (07) 3138 5123 or email ethicscontact@qut.edu.au. The QUT Research Ethics Unit is not connected with the research project and can facilitate a resolution to your concern in an impartial manner.

**About this survey**

This survey is designed to understand your beliefs about dieting, healthy eating, and weight control.

Your answers to the questions are confidential and anonymous.

For each question, please circle the number that represents your opinion. Some questions may seem very similar, but they are different and we would like you to answer all of them if possible.

Thank you for your help

**Background information**

**What is your age? ___________**

**Are you male or female?**

| Male  Female |
| --- |

**What is your current marital status?**

| Married  De facto  Separated  Divorced  Widowed  Never married  Don’t know |
| --- |

**What is your employment status?**

| Employed, full-time  Employed part-time or casual  Home duties or carer  Unemployed  Full-time student  Part-time student  Retired  Permanently ill / unable to work  Prefer not to answer |
| --- |
| Other (please specify) |

**We are interested in how income relates to health and lifestyle.

Before tax is taken out, which of the following ranges best describes your household’s income, from all sources, over the last 12 months?**

| Less than $20,000  $20,001 - $30,000  $30,001 - $50,000  $50,001 - $100,000  $100,001 - $150,000  Over $150,000  Don’t know  Prefer not to answer |
| --- |

**So we can accurately establish the boundaries of our survey areas for statistical purposes, may we ask what postcode you live in?**

**What is the name of the suburb, town or community that you live in?**

**How tall are you (in centimetres) without shoes on?**

**What is your weight in kilograms (kgs)?**

**Are you required to diet for any reason?**

| Yes  No |
| --- |
| If you are required to diet, please list the reason: |

**IMPORTANT: your code identifier

This is used to anonymously match up your response today with your response 4 weeks later**

| What is the first letter of your first name? |  |
| --- | --- |
| What is the third letter of your first name? |  |
| What is the first letter in your mother’s first name? |  |
| What date of the month were you born on? (e.g., 24th) |  |

For example, imagine Louise Smith is filling out the survey.
Her mother's name is Anne.
Louise was born on the 31st December.

Louise's code identifier would be:
L
U
A
31

**IMPORTANT: What does dieting mean?**

In this survey, when we talk about ‘dieting’, we mean:

**Intentionally restricting your calorie intake (energy or kilojoules derived from food) or increasing certain type of foods (e.g., high carbohydrate or high protein) to lose weight and/or change your body shape.**

This does not include fasting for religious purposes.

This includes any eating program planned by someone else, or any approach you have designed on your own, used in any context, to lose weight and/or change your shape. Examples include:

- Popular diets (e.g., The Paleo Diet or The Pritikin Diet)
- Commercial weight loss programs (e.g., Weight Watchers or Jenny Craig)
- Meal replacements or supplements (e.g., Tony Ferguson or OptiSlim)

Anti-dieting subscale of the Intuitive Eating Scale (Hawks, Merrill, & Madanat, 2004):

**Please select the response which best represents your opinion for each statement**

|  | Strongly disagree | Disagree | Neutral | Agree | Strongly agree |
| --- | --- | --- | --- | --- | --- |
| I generally count calories before deciding if something is OK to eat |  |  |  |  |  |
| One of my main reasons for exercising is to manage my weight |  |  |  |  |  |
| I am hopeful that I will someday find a new diet that will actually work for me |  |  |  |  |  |
| There are certain foods that I really like, but I try to avoid them so that I won’t gain weight |  |  |  |  |  |
| I am often frustrated with my body size and wish that I could control it better |  |  |  |  |  |
| I am afraid to be around some foods because I don’t want to be tempted to indulge myself |  |  |  |  |  |
| I am often either on a diet or seriously considering going on a diet |  |  |  |  |  |
| I usually feel like a failure when I eat more than I should |  |  |  |  |  |
| I often feel physically weak and hungry because I am dieting to control my weight |  |  |  |  |  |
| I often put off buying clothes, participating in fun activities, or going on vacations (hoping I can get thinner first) |  |  |  |  |  |
| I feel pressure from those around me to control my weight or watch what I eat |  |  |  |  |  |
| I worry more about how fattening a food might be, rather than how nutritious it might be |  |  |  |  |  |
| I feel safest if I have a diet plan, or diet menu, to guide my eating |  |  |  |  |  |

Past dieting year:

**Remember, dieting is the intentional restriction of your calorie intake (energy or kilojoules derived from food) or increasing types of foods (e.g., high carbohydrate or high protein) to lose weight and/or change your body shape.**

| How many times have you dieted in the past year? |  |
| --- | --- |

Past dieting and non-dieting:

**Please select the response which best represents your opinion for each statement**

|  | No, definitely not | 2 | 3 | Neutral | 5 | 6 | Yes, definitely |
| --- | --- | --- | --- | --- | --- | --- | --- |
| I dieted in the **past month** |  |  |  |  |  |  |  |
| I ate healthily without dieting in the **past month** |  |  |  |  |  |  |  |

Time 1 dieting and non-dieting behaviour:

**These questions are about what you are currently doing**

|  | No, definitely not | 2 | 3 | Neutral | 5 | 6 | Yes, definitely |
| --- | --- | --- | --- | --- | --- | --- | --- |
| I am **currently** dieting |  |  |  |  |  |  |  |
| If you said yes, please list what strategies or approaches you used | | | | | | | |
| I am **currently** eating healthily without dieting |  |  |  |  |  |  |  |
| If you said yes, please list what strategies or approaches you  used | | | | | | | |

Dieting and non-dieting intentions:

**Please select the response which best represents your opinion**

|  | No, definitely not | 2 | 3 | Neutral | 5 | 6 | Yes, definitely |
| --- | --- | --- | --- | --- | --- | --- | --- |
| I intend to diet in the **next month** |  |  |  |  |  |  |  |
| I intend to eat healthily without dieting in the **next month** |  |  |  |  |  |  |  |

Weight control beliefs questionnaire (Laliberte, Newton, McCabe, & Mills):

**Please select the response that best represents your opinion**

|  | Not at all true | 2 | 3 | Somewhat true | 5 | 6 | Very true |
| --- | --- | --- | --- | --- | --- | --- | --- |
| I believe I should control my weight |  |  |  |  |  |  |  |
| I try to live a healthy lifestyle and let my weight go to what is natural for me |  |  |  |  |  |  |  |
| I focus on healthy living rather than on controlling my weight |  |  |  |  |  |  |  |
| If I work at it, I should be able to keep my weight where I want it |  |  |  |  |  |  |  |
| I try to accept the weight that is natural for me and focus on living a healthy lifestyle |  |  |  |  |  |  |  |
| If I stick to the right exercise and eating plan, I should be able to achieve the weight and shape I want |  |  |  |  |  |  |  |
| If I am living a healthy lifestyle, my body is likely at the weight I am meant to be |  |  |  |  |  |  |  |
| It is important to me that I accept the weight that comes with living a healthy lifestyle |  |  |  |  |  |  |  |
| The main thing that determines my weight is what I myself do |  |  |  |  |  |  |  |
| If I am careful, I can control my weight |  |  |  |  |  |  |  |
| I’d rather live healthily and accept that we all come in different shapes and sizes |  |  |  |  |  |  |  |
| If my weight is more than I want it to be, then I am at fault |  |  |  |  |  |  |  |
| Whether I gain, lose or maintain my weight is within my control |  |  |  |  |  |  |  |
| I am comfortable letting my weight fluctuate naturally |  |  |  |  |  |  |  |
| I focus on healthy eating rather than trying to control my weight |  |  |  |  |  |  |  |
| If I want to be a certain weight, I can make it happen |  |  |  |  |  |  |  |
| I focus on healthy exercise rather than trying to control my weight |  |  |  |  |  |  |  |

Attribution for dieting failure:

**If I was not able to follow a diet, it would be**

| My fault | 2 | 3 | My fault and the diet's fault | 5 | 6 | The diet's fault |
| --- | --- | --- | --- | --- | --- | --- |
|  |  |  |  |  |  |  |

Attitudes towards dieting:

**For the questions below, please answer each question. 

Dieting in the next month would be...**

| Harmful | 2 | 3 | Neutral | 5 | 6 | Beneficial |
| --- | --- | --- | --- | --- | --- | --- |
|  |  |  |  |  |  |  |

**Dieting in the next month would be...**

| Pleasant | 2 | 3 | Neutral | 5 | 6 | Unpleasant |
| --- | --- | --- | --- | --- | --- | --- |
|  |  |  |  |  |  |  |

**Dieting in the next month would be...**

| Foolish | 2 | 3 | Neutral | 5 | 6 | Wise |
| --- | --- | --- | --- | --- | --- | --- |
|  |  |  |  |  |  |  |

**Dieting in the next month would be...**

| Bad | 2 | 3 | Neutral | 5 | 6 | Good |
| --- | --- | --- | --- | --- | --- | --- |
|  |  |  |  |  |  |  |

**Dieting in the next month would be...**

| Ineffective | 2 | 3 | Neutral | 5 | 6 | Effective |
| --- | --- | --- | --- | --- | --- | --- |
|  |  |  |  |  |  |  |

Subjective norms for dieting:

**Most people who are important to me think that I...**

| Should not diet | 2 | 3 | Neutral | 5 | 6 | Should diet |
| --- | --- | --- | --- | --- | --- | --- |
|  |  |  |  |  |  |  |

**Please select the response which best reflects your opinion**

|  | No, definitely not | 2 | 3 | Neutral | 5 | 6 | Yes, definitely |
| --- | --- | --- | --- | --- | --- | --- | --- |
| It is expected of me that I will diet |  |  |  |  |  |  |  |
| People who are important to me diet |  |  |  |  |  |  |  |

Expectations of dieting:

**If I dieted in the next month, I would**

| Lose weight | 2 | 3 | Weigh the same | 5 | 6 | Gain weight |
| --- | --- | --- | --- | --- | --- | --- |
|  |  |  |  |  |  |  |

**If I dieted in the next month, I would**

| Get thicker or fatter | 2 | 3 | Stay the same | 5 | 6 | Get slimmer or thinner |
| --- | --- | --- | --- | --- | --- | --- |
|  |  |  |  |  |  |  |

Self-efficacy to diet:

**How confident are you that you would be able to diet in the next month?**

| Not confident at all | 2 | 3 | Neutral | 5 | 6 | Very confident |
| --- | --- | --- | --- | --- | --- | --- |
|  |  |  |  |  |  |  |

**I believe I have the ability to diet in the next month**

| No, definitely not | 2 | 3 | Neutral | 5 | 6 | Yes, definitely |
| --- | --- | --- | --- | --- | --- | --- |
|  |  |  |  |  |  |  |

Perceived control over dieting:

**The decision to diet in the next month is beyond my control**

| Strongly disagree | 2 | 3 | Neutral | 5 | 6 | Strongly agree |
| --- | --- | --- | --- | --- | --- | --- |
|  |  |  |  |  |  |  |

**Whether or not I diet in the next month is entirely up to me**

| Strongly disagree | 2 | 3 | Neutral | 5 | 6 | Strongly agree |
| --- | --- | --- | --- | --- | --- | --- |
|  |  |  |  |  |  |  |

Non-planning subscale (Spinella, 2007)

**For each item, please select the response which best reflects your opinion**

|  | Rarely/never | 2 | 3 | Almost always/always |
| --- | --- | --- | --- | --- |
| I plan for job security |  |  |  |  |
| I plan for the future |  |  |  |  |
| I save regularly |  |  |  |  |
| I plan tasks carefully |  |  |  |  |
| I am a careful thinker |  |  |  |  |

Self-identity as a non-dieter (first three items) and dieter (last three items)

**For each item, please select the response which best reflects your opinion**

|  | No, definitely not | 2 | 3 | Neutral | 5 | 6 | Yes, definitely |
| --- | --- | --- | --- | --- | --- | --- | --- |
| I think of myself as a healthy eater |  |  |  |  |  |  |  |
| I think of myself as a normal weight person |  |  |  |  |  |  |  |
| I think of myself as someone who eats healthily without dieting |  |  |  |  |  |  |  |
| I think of myself as someone who is concerned with my weight |  |  |  |  |  |  |  |
| I think of myself as a dieter |  |  |  |  |  |  |  |
| I think of myself as an overweight person |  |  |  |  |  |  |  |

Body Image Acceptance and Action Questionnaire (Sandoz, Wilson, Merwin, & Kate Kellum, 2013)

**Please select the response which best reflects your opinion**

|  | Never true | 2 | 3 | Neutral | 5 | 6 | Always true |
| --- | --- | --- | --- | --- | --- | --- | --- |
| Worrying about my weight makes it difficult for me to live a life that I value |  |  |  |  |  |  |  |
| I care too much about my weight and body shape |  |  |  |  |  |  |  |
| I shut down when I feel bad about my body shape or weight |  |  |  |  |  |  |  |
| My thoughts and feelings about my body weight and shape must change before I can take important steps in my life |  |  |  |  |  |  |  |
| Worrying about my body takes up too much of my time |  |  |  |  |  |  |  |
| If I start to feel fat, I try to think about something else |  |  |  |  |  |  |  |
| Before I can make any serious plans, I have to feel better about my body |  |  |  |  |  |  |  |
| I will have better control over my life if I can control my negative thoughts about my body |  |  |  |  |  |  |  |
| To control my life, I need to control my weight |  |  |  |  |  |  |  |
| Feeling fat causes problems in my life |  |  |  |  |  |  |  |
| When I start thinking about the size and shape of my body, it’s hard to do anything else |  |  |  |  |  |  |  |
| My relationships would be better if my body weight and/or shape did not bother me |  |  |  |  |  |  |  |

Intuitive Eating Scale-2 (Tylka & Kroon Van Diest, 2013):

**Please select the response which best reflects your opinion**

|  | Strongly disagree | 2 | 3 | Neutral | 5 | 6 | Strongly agree |
| --- | --- | --- | --- | --- | --- | --- | --- |
| I try to avoid certain foods high in fat, carbohydrates, or calories |  |  |  |  |  |  |  |
| I find myself eating when I’m feeling emotional (e.g., anxious, depressed, sad), even when I’m not physically hungry |  |  |  |  |  |  |  |
| If I am craving a certain food, I allow myself to have it |  |  |  |  |  |  |  |
| I find myself eating when I am lonely, even when I’m not physically hungry |  |  |  |  |  |  |  |
| I get mad at myself for eating something unhealthy |  |  |  |  |  |  |  |
| I trust my body to tell me **when** to eat |  |  |  |  |  |  |  |
| I trust my body to tell me **what** to eat |  |  |  |  |  |  |  |
| I trust my body to tell me **how much** to eat |  |  |  |  |  |  |  |
| I have forbidden foods that I don’t allow myself to eat |  |  |  |  |  |  |  |
| I use food to help me soothe my negative emotions |  |  |  |  |  |  |  |
| I find myself eating when I am stressed out, even when I’m not physically hungry |  |  |  |  |  |  |  |
| I am able to cope with my negative emotions (e.g., anxiety, sadness) without turning to food for comfort |  |  |  |  |  |  |  |
| When I am bored, I do NOT eat just for something to do |  |  |  |  |  |  |  |
| When I am lonely, I do NOT turn to food for comfort |  |  |  |  |  |  |  |
| I find other ways to cope with stress and anxiety than by eating |  |  |  |  |  |  |  |
| I allow myself to eat what food I desire at the moment |  |  |  |  |  |  |  |
| I do NOT follow eating rules or dieting plans that dictate what, when, and/or how much to eat |  |  |  |  |  |  |  |
| Most of the time, I desire to eat nutritious foods |  |  |  |  |  |  |  |
| I mostly eat foods that make my body perform efficiently (well) |  |  |  |  |  |  |  |
| I mostly eat foods that give my body energy and stamina |  |  |  |  |  |  |  |
| I rely on my hunger signals to tell me when to eat |  |  |  |  |  |  |  |
| I rely on my fullness (satiety) signals to tell me when to stop eating |  |  |  |  |  |  |  |
| I trust my body to tell me when to stop eating |  |  |  |  |  |  |  |

Attitudes towards non-dieting:

**For the questions below, please answer each question. 

Eating healthily without dieting in the next month would be...**

| Harmful | 2 | 3 | Neutral | 5 | 6 | Beneficial |
| --- | --- | --- | --- | --- | --- | --- |
|  |  |  |  |  |  |  |

**Eating healthily without dieting in the next month would be...**

| Pleasant | 2 | 3 | Neutral | 5 | 6 | Unpleasant |
| --- | --- | --- | --- | --- | --- | --- |
|  |  |  |  |  |  |  |

**Eating healthily without dieting in the next month would be...**

| Foolish | 2 | 3 | Neutral | 5 | 6 | Wise |
| --- | --- | --- | --- | --- | --- | --- |
|  |  |  |  |  |  |  |

**Eating healthily without dieting in the next month would be...**

| Bad | 2 | 3 | Neutral | 5 | 6 | Good |
| --- | --- | --- | --- | --- | --- | --- |
|  |  |  |  |  |  |  |

**Eating healthily without dieting in the next month would be...**

| Ineffective | 2 | 3 | Neutral | 5 | 6 | Effective |
| --- | --- | --- | --- | --- | --- | --- |
|  |  |  |  |  |  |  |

Subjective norms for non-dieting:

**Most people who are important to me think that I...**

| Should not eat healthily without dieting | 2 | 3 | Neutral | 5 | 6 | Should eat healthily without dieting |
| --- | --- | --- | --- | --- | --- | --- |
|  |  |  |  |  |  |  |

**Please select the response which best reflects your opinion**

|  | No, definitely not | 2 | 3 | Neutral | 5 | 6 | Yes, definitely |
| --- | --- | --- | --- | --- | --- | --- | --- |
| It is expected of me that I will eat healthily without dieting |  |  |  |  |  |  |  |
| People who are important to me eat healthily without dieting |  |  |  |  |  |  |  |

Non-dieting self-efficacy:

**How confident are you that you will be able to eat healthily without dieting in the next month?**

| Not confident at all | 2 | 3 | Neutral | 5 | 6 | Very confident |
| --- | --- | --- | --- | --- | --- | --- |
|  |  |  |  |  |  |  |

**I believe I have the ability to eat healthily without dieting in the next month**

| No, definitely not | 2 | 3 | Neutral | 5 | 6 | Yes, definitely |
| --- | --- | --- | --- | --- | --- | --- |
|  |  |  |  |  |  |  |

Non-dieting perceived control:

**The decision to eat healthily without dieting in the next month is beyond my control**

| Strongly disagree | 2 | 3 | Neutral | 5 | 6 | Strongly agree |
| --- | --- | --- | --- | --- | --- | --- |
|  |  |  |  |  |  |  |

**Whether or not I eat healthily without dieting in the next month is entirely up to me**

| Strongly disagree | 2 | 3 | Neutral | 5 | 6 | Strongly agree |
| --- | --- | --- | --- | --- | --- | --- |
|  |  |  |  |  |  |  |

**Thank you very much for your time!

If you know of any males who may be interested in participating in this research, please feel free to send the information sheet or link onto them.

Follow-up survey

IMPORTANT: You have the option to participate in a short 7-item (approximately 5 minutes) follow-up survey in 4 weeks’ time. If you are willing to be contacted for this follow-up survey, could you please provide your contact details in the section below?

Your contact details will NOT be used for any other purpose and the only person who will have access to your details will be the primary researcher. These contact details will be deleted once you have completed the follow-up survey. We will be able to link your responses for the two surveys using the anonymous code you generated. Please provide your details if you wish to complete the short follow-up survey.**

| Today's date: |  |
| --- | --- |
| Your first name: |  |
| If you would like to receive the second survey online, please provide your e-mail address: |  |
| If you would like to complete the second survey over the phone, please provide your phone number and best contact time (day/night, weekday/weekend): |  |

**Prize Draw

To go into the prize draw to win one of three iPad Minis, please provide your first name and contact number below. These details will be separated from your questionnaire responses and will not be used for any other purpose. Please note that if you complete the second survey in a month’s time you will be able to enter this prize draw again. This prize draw will be conducted on Friday 20th December, 2013.**

| Name: |  |
| --- | --- |
| Best contact number: |  |

**Research Results

If you wish to receive a summary of the research results once the study has been completed, please provide your contact details below. These details will be stored separately from the completed survey.

Please specify email or postal mail address:**

Study 3 Time 2 questionnaire

**Dieting, healthy eating, and weight control – follow-up questionnaire**

**About this survey**

This survey is the follow-up survey to the survey you filled in 1 month ago. This survey involves a very small subset of the questions you completed a month ago.

Your answers to the questions are confidential and anonymous.

**Important: Your code identifier

This is used to anonymously match up your response today with your response 4 weeks ago**

| What is the first letter of your first name? |  |
| --- | --- |
| What is the third letter of your first name? |  |
| What is the first letter in your mother’s first name? |  |
| What is the date of the month you were born on? (e.g., 24th) |  |

For example, imagine Louise Smith is filling out the survey.
Her mother’s name is Anne. 
Louise was born on the 31st of December. 

Her code identifier would be: 
L
U
A
31

**IMPORTANT: What does dieting mean?**

In this survey, when we talk about ‘dieting’, we mean:

**Intentionally restricting your calorie intake (energy or kilojoules derived from food) or increasing certain type of foods (e.g., high carbohydrate or high protein) to lose weight and/or change your body shape.**

This does not include fasting for religious purposes.

This includes any eating program planned by someone else, or any approach you have designed on your own, used in any context, to lose weight and/or change your shape. Examples include:

- Popular diets (e.g., The Paleo Diet or The Pritikin Diet)
- Commercial weight loss programs (e.g., Weight Watchers or Jenny Craig)
- Meal replacements or supplements (e.g., Tony Ferguson or OptiSlim)

Time 2 behaviour:

**Please circle the number below that best represents your response**

|  | No, definitely not | 2 | 3 | Neutral | 5 | 6 | Yes, definitely |
| --- | --- | --- | --- | --- | --- | --- | --- |
| I went on a diet **in the past month** |  |  |  |  |  |  |  |
| If you said yes, please list what strategies or approaches you used | | | | | | | |
| I ate healthily without dieting **in the past month** |  |  |  |  |  |  |  |
| If you said yes, please list what strategies or approaches you used | | | | | | | |

**What is your definition of dieting?**

1-item measures to establish validity of time 2 dependent variables:

**Please think of the previous month when answering the following:**

|  | No, definitely not | 2 | 3 | Neutral | 5 | 6 | Yes, definitely |
| --- | --- | --- | --- | --- | --- | --- | --- |
| I changed my eating to **lose weight** in the past month |  |  |  |  |  |  |  |
| I changed my eating to **alter my body shape** in the past month |  |  |  |  |  |  |  |
| I **restricted my calorie intake** in the past month |  |  |  |  |  |  |  |
| I **ate healthily** in the past month |  |  |  |  |  |  |  |

**Thank you very much for your time!

To add another entry into the prize draw to win one of 3 iPad minis, please provide your first name and contact number below. These details will be separated from your questionnaire responses and will not be used for any other purpose. This prize draw will be conducted on Friday 20th December, 2013.**

| Name: |  |
| --- | --- |
| Best contact number: |  |
